# Early predictive value of ultrasound measurements of rectus femoris cross-sectional area to diagnose ICU-acquired weakness in patients undergoing invasive mechanical ventilation: a prospective cohort study

**DOI:** 10.1186/s40001-024-01966-6

**Published:** 2024-07-20

**Authors:** Huiming Yao, Jie Zhang, Rong Jiang, Qian Xie, Chaoqi Zhou, Yuting Yang, Zhenguo Zeng, Wei Zhang

**Affiliations:** 1 Department of Critical Care Medicine, Medical Center of Anesthesiology and Pain, The First Affiliated Hospital, Jiangxi Medical College, Nanchang University, Nanchang, 330006 China; 2 Department of Respiratory and Critical Care Medicine, The First Affiliated Hospital, Jiangxi Medical College, Nanchang University, Nanchang, 330006 China; 3 Jiangxi Provincial Key Laboratory of Respiratory Diseases, The First Affiliated Hospital, Jiangxi Medical College, Nanchang University, Nanchang, 330006 China

**Keywords:** ICU-acquired weakness, Ultrasound measurements, Rectus femoris cross-sectional area, Mechanical ventilation, Medical Research Council\

## Abstract

**Background:**

The diagnosis of ICU-acquired weakness (ICUAW) may be delayed due to the complexity of critically ill patients. This study aimed to investigate the value of ultrasound measurements of rectus femoris cross-sectional area (RFCSA) in predicting ICUAW in patients undergoing invasive mechanical ventilation.

**Methods:**

This was a prospective cohort study of patients undergoing mechanical ventilation for at least 48 h. RFCSA was measured using ultrasound in patients upon ICU admission and followed until discharge. Using the Medical Research Council score as the gold standard, we evaluated the diagnostic value of ultrasound measurements in predicting ICUAW. Kaplan–Meier curves were constructed to evaluate and compare the length of ICU stay and duration of invasive mechanical ventilation between patients with and without ICUAW.

**Results:**

Among the 76 patients, 34 (44.7%) were diagnosed with ICUAW using the Medical Research Council score as the gold standard. The RFCSA atrophy rate between day 1 and day 3 was significantly higher in the ICUAW group (7.9 ± 2.8% vs. 4.3 ± 2.1%, *p* < 0.001). By utilizing a cutoff point of 6.9%, we discovered that the RFCSA atrophy rate exhibited excellent diagnostic accuracy in predicting ICUAW, with a sensitivity of 76.5% and specificity of 92.9%. In ICUAW patients diagnosed based on an RFCSA atrophy rate, the proportion of patients with an ICU stay longer than 14 days was 42.9%, which was significantly higher compared to 22.9% in the non-ICUAW group (HR: 1.768; 95% CI 1.128–2.772; *p* = 0.006). Similarly, the proportion of patients continuing mechanical ventilation at 14 days was 28.6% versus 4.2% between the two groups (HR: 1.988; 95% CI 1.266–3.120; p < 0.001).

**Conclusion:**

Ultrasound measurements of RFCSA provide a reliable method for diagnosing ICUAW and indicating prognosis in patients undergoing invasive mechanical ventilation.

## Background

Neuromuscular weakness can develop in the intensive care unit (ICU) after hours of invasive mechanical ventilation (MV) and persist for years, resulting in long-term dysfunction [1]. This weakness, known as ICU-acquired weakness (ICUAW), is a secondary disorder that occurs while patients are being treated for other life-threatening conditions [2]. ICUAW is characterized by clinically detected weakness without any identifiable cause other than critical illness and its treatments [3]. It is a significant contributor to morbidity among critically ill patients, affecting approximately 25% of those on MV [4]. Consequently, it is estimated that over 75,000 patients in the United States and up to 1 million people worldwide may develop a generalized frailty syndrome referred to as ICUAW [5]. Patients with ICUAW experience severe weakness in their extremities and often struggle to be weaned off the ventilator. The condition encompasses various neuromuscular disturbances, including “critical illness polyneuropathy”, “critical illness myopathy”, or a combination of both known as “critical illness neuromyopathy”. Clinical diagnosis of ICUAW involves limb paresis but can also involve respiratory muscles, leading to changes in inspiratory and expiratory force, as well as pharyngeal muscles, commonly resulting in overall respiratory muscle weakness and swallowing disorders [6–8].

Given the complexity of critically ill patients, both standard definitions and disease-classification features for ICUAW are challenging to establish and apply in clinical practice. Currently, electrophysiological and nerve conduction studies remain the most accurate methods for diagnosing ICUAW. These studies can detect electrophysiological changes as early as 24 to 48 h after the onset of ICUAW, often preceding clinical findings [9, 10]. Sural and peroneal nerve conduction studies show promise as reliable screening tools, potentially assisting in the identification of patients who require further confirmation of the diagnosis [11]. However, electrophysiological methods, such as electroneurography and electromyography, are reported to be used by 32.6% of study participants, suggesting significant barriers in clinical practice [12]. Performing electrophysiological and nerve conduction studies in critically ill patients is not straightforward. The former requires patients to be awake and able to contract muscles voluntarily, which is often challenging for critically ill patients. Moreover, issues such as tissue edema and muscle necrosis can confound nerve conduction studies [7]. Given its practicality, the predominant selective assessment tool employed in diagnosing ICUAW is the Medical Research Council (MRC) scale, which assesses the strength of various muscle groups in the upper and lower extremities. Scores range from 0 to 5, with higher scores indicating greater muscle strength. A composite score below 48 is diagnostic of ICUAW, while scores below 36 indicate severe ICUAW [7, 13, 14]. In theory, assessing muscle strength to determine the distribution and extent of ICUAW is practical. However, during early critical illness, a significant proportion of patients are deeply sedated while receiving invasive MV, and only approximately 25 to 29% of patients are awake enough to undergo muscle strength assessment [13, 15]. Consequently, the diagnosis of ICUAW may be delayed, highlighting the need for more accurate methods to evaluate muscle strength.

Critically ill patients experience significant and rapid muscle wasting during their ICU stay. Research has shown that a considerable loss of muscle mass, exceeding 10% within the first week in the ICU, is associated with functional impairment [16, 17]. The decline in lean body mass has been clinically linked to adverse patient outcomes [18]. The catabolic state induced by critical illness, characterized by decreased anabolic hormones and increased catabolic hormones, combined with mechanical unloading from immobilization and denervation, contributes to muscle atrophy and the development of ICUAW in critically ill patients [2, 19]. Assessment of the quadriceps femoris muscle is considered valuable, as it serves as a reliable indicator of muscle strength [20]. Compared to other muscle groups, the quadriceps femoris muscle holds particular importance and has demonstrated good correlation with skeletal muscle mass when examined using ultrasound measurements. This technique exhibits optimal intraobserver and interobserver reliability [18]. Skeletal muscle ultrasound serves as a diagnostic and risk stratification tool for evaluating skeletal muscle dysfunction [21]. It enables the identification of structural and shape changes in muscles and allows for monitoring muscle alterations over time [22]. In critically ill patients requiring invasive MV, ultrasound offers several advantages, including noninvasiveness, repeatability at the bedside, and the absence of a requirement for patient cooperation.

The rapid decline in muscle strength leads to muscle atrophy and the development of ICUAW. Despite practical and ethical challenges that hinder researchers' understanding of the disease mechanisms, studies involving patients and animal models have provided valuable insights into ICUAW, attributing it to complex structural changes within muscles [6, 23]. In contrast to the muscle wasting observed in chronic unloading conditions, critically ill patients experience a marked and preferential loss of myosin and myosin-associated proteins [24]. Observational studies have reported that muscle wasting is associated with a prolonged duration of ICU stay [25] and higher ICU and hospital mortality rates [26], indicating the clinical importance of this condition throughout different phases of critical illness, from acute to recovery. While there has been growing interest in studying muscle wasting and using point-of-care ultrasound measurements of the quadriceps muscle in critically ill patients [20, 27], few studies have focused on assessing the role of ultrasound measurements in diagnosing ICUAW specifically in patients undergoing invasive MV during their ICU stay [28]. Therefore, the primary aim of this study was to evaluate the ultrasound characteristics of the rectus femoris, a component of the quadriceps muscle, and examine their variations throughout the ICU stay. As secondary objectives, we aimed to investigate the potential of ultrasound measurements in predicting ICUAW and indicating prognosis at an earlier stage compared to traditional MRC scoring, which is typically performed when patients are able to cooperate.

## Methods

### Patients

This monocentric prospective cohort study was conducted at the mixed medical-surgical ICU of The First Affiliated Hospital, Jiangxi Medical College, Nanchang University in Nanchang, China, and was designed in accordance with the STARD criteria [29]. Informed consent was obtained from all patients or their legal representatives. As a regional critical care center, ultrasound evaluation was a part of routine medical and nursing care. All patients could receive a standardized ultrasound evaluation at the moment they were admitted to the ICU. Only patients newly admitted to ICU and receiving invasive MV for more than 48 h were eligible for inclusion. Patients were excluded if they were pregnant, aged below 18 years, had a lower-limb amputation, had limb weakness prior to ICU admission, had a primary neuromuscular pathology (such as stroke, lower-limb immobilization from a plaster, neurodegenerative diseases, peripheral neuropathy or muscle dystrophy), or had terminal cancer. Patients who did not receive invasive MV when admitted to ICU but underwent endotracheal intubation after a period of treatment were not included in this study.

### Clinical data collection

Clinical factors that have been previously reported in the literature to potentially influence the onset of ICUAW were selected and recorded for eligible patients [30]. These factors included potential risk factors present at ICU admission, primary diagnosis in intensive care, enteral nutrition data, Acute Physiology and Chronic Health Evaluation II (APACHE II) score, presence of sepsis (according to the sepsis 3.0 criteria) [31], and drug therapy involving sedatives, vasopressors, and parenteral nutrition. Regarding medications suspected of affecting episodes of ICUAW, we documented whether patients received these drugs at least once after their ICU admission.

### Medical Research Council score

The enrolled patients were assessed daily for their level of cooperation based on their facial responses to five specific orders: “Open (close) your eyes”, “Look at me”, “Open your mouth and put out your tongue”, “Nod your head”, and “Raise your eyebrows when I count up to 5.” Patients who responded to at least three of these commands during two consecutive evaluations were considered cooperative. Muscle strength assessment was performed as soon as patients reached a satisfactory level of wakefulness (Richmond Agitation Sedation Scale between − 1 and 1) and demonstrated cooperation. To minimize interexaminer variability, all researchers involved in the study underwent comprehensive training in measuring the MRC score. In this study, three examiners were involved in MRC scoring. Each examiner underwent a minimum of 2 weeks of theoretical training on MRC scoring before engaging in the study. To ensure accuracy during the scoring process, if an examiner could not assure the precision of the scoring outcomes, another examiner was called upon for a secondary assessment. The average of the two scores was then calculated to reduce discrepancies and enhance accuracy. The MRC score is a validated scoring system that evaluates the strength of six muscle groups on each side of the body: shoulder abductors, elbow flexors, wrist extensors, hip flexors, knee extensors, and ankle dorsiflexors. Each muscle group is assigned a score ranging from 0 (no visible contraction) to 5 (normal force throughout the complete range of motion). The total MRC score ranges from 0 to 60. In accordance with previously published literature, ICUAW is defined as an MRC score below 48, while a score below 36 indicates severe ICUAW [14].

### Image acquisition and ultrasound measurements of RFCSA

**Fig. 1:**
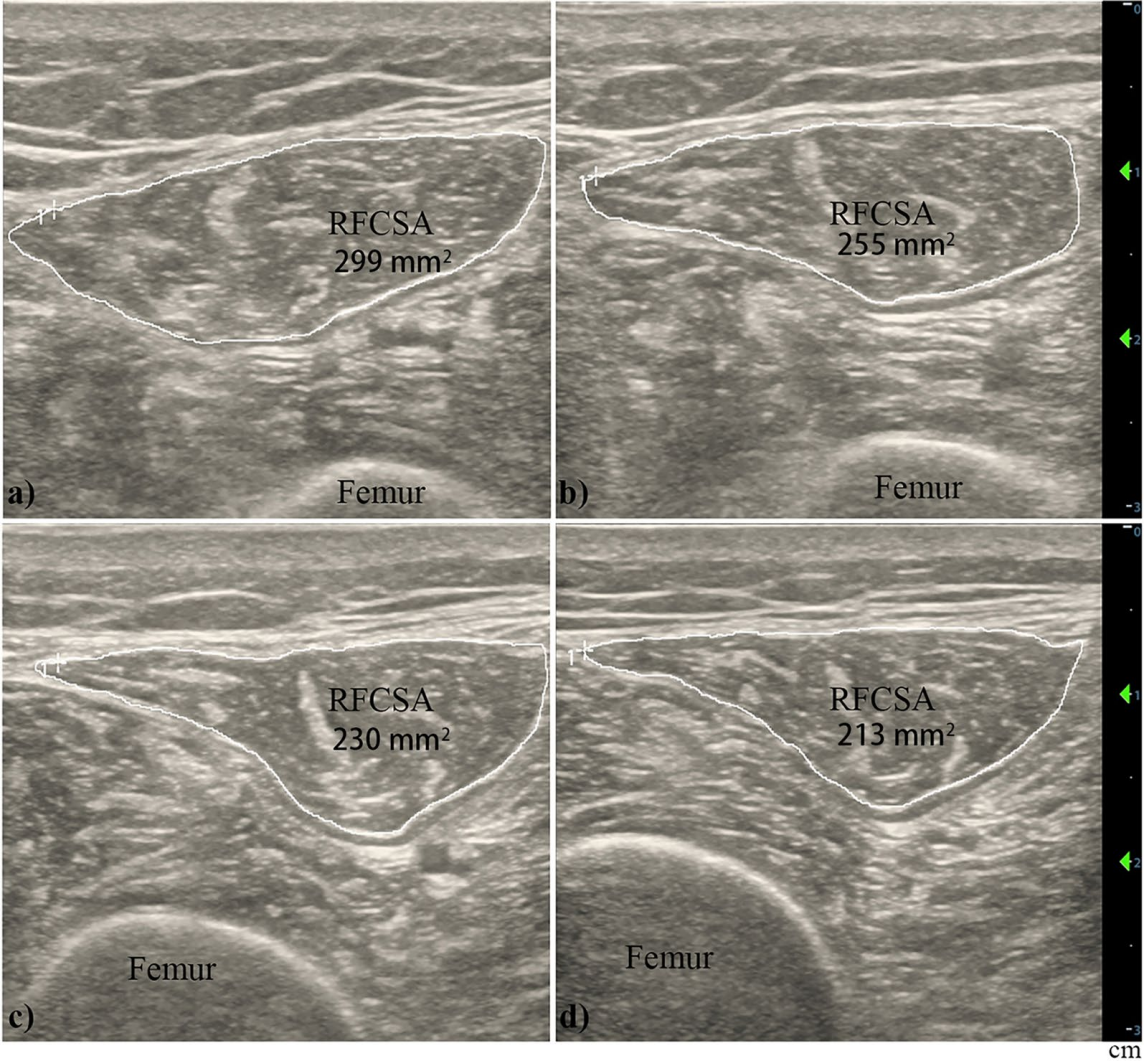
Ultrasound measurements of RFCSA among patients undergoing invasive mechanical ventilation. The RFCSA is outlined by a white border in each image. RFCSA on D1 of ICU admission (**a**), RFCSA on D3 (**b**), RFCSA on D5 (**c**), and RFCSA on D7 (**d**). The gain and depth settings were consistent across all images. RFCSA, rectus femoris cross-sectional area

Ultrasound measurements of the rectus femoris cross-sectional area (RFCSA) were performed as part of routine clinical monitoring carried out in the unit. To image the rectus femoris, the patient was positioned at 30 degrees on a suitable bed. The knee was relaxed to prevent artificial contraction. B-mode ultrasonography using an 8 MHz, 4 cm linear probe (Mindray M9 machine, Mindray BioMedical Electronics Co. Ltd. Shenzhen, China) was placed on the anterior thigh at a predetermined point, which was two-thirds of the distance from the anterior superior iliac spine to the upper border of the patella. This positioning allowed for visualization of various structures, including subcutaneous tissues, adipose tissue, the quadriceps femoris muscle (including the rectus femoris, vastus medialis, vastus intermedius, and vastus lateralis), and the femur. Once the muscle tissue was identified, RFCSA and other characteristics of the femur were recorded by a trained operator who remained blinded to subsequent muscle strength assessments. Figure 1 illustrates the repeated measurements taken on the right leg on day 1 (D1), day 3 (D3), day 5 (D5), and day 7 (D7) after ICU admission. Three measurements were taken for each data point, and the average value was used for further analysis. If the maximum difference between the three data exceeded 20%, another researcher with ultrasound qualifications was invited to take measurements again, and the average of the data obtained by the two researchers was taken.

### Statistical analysis

Continuous variables were presented as the mean ± standard deviation (SD). Univariate analyses involved the use of independent t-tests or the Mann–Whitney U test according to their distribution to compare continuous variables, while categorical variables were compared using Chi-square tests or Fisher's exact tests. Based on the data distribution, correlations between variables demonstrating statistical variances and the outcome variable were calculated utilizing Spearman or Pearson correlation coefficients. Using MRC score as the gold standard, we evaluated the diagnostic value of muscle ultrasound in predicting ICUAW through receiver operating characteristic (ROC) curve analysis. The optimal cutoff value of the ROC curve was established using the Youden Index method. Kaplan–Meier curves were constructed to assess the duration of ICU stay and invasive MV between patients with and without ICUAW. The log-rank test was employed to compare patients with ICUAW to those without ICUAW. Hazard ratios (HR) with corresponding 95% confidence intervals (CI) were calculated for clinical factors. Statistical analyses were performed using SPSS version 23, and R statistical software version 4.1.3 was used for specific analyses. A p-value less than 0.05 was considered statistically significant.

## Results

### Flow diagram of patient screening and inclusion

**Fig. 2:**
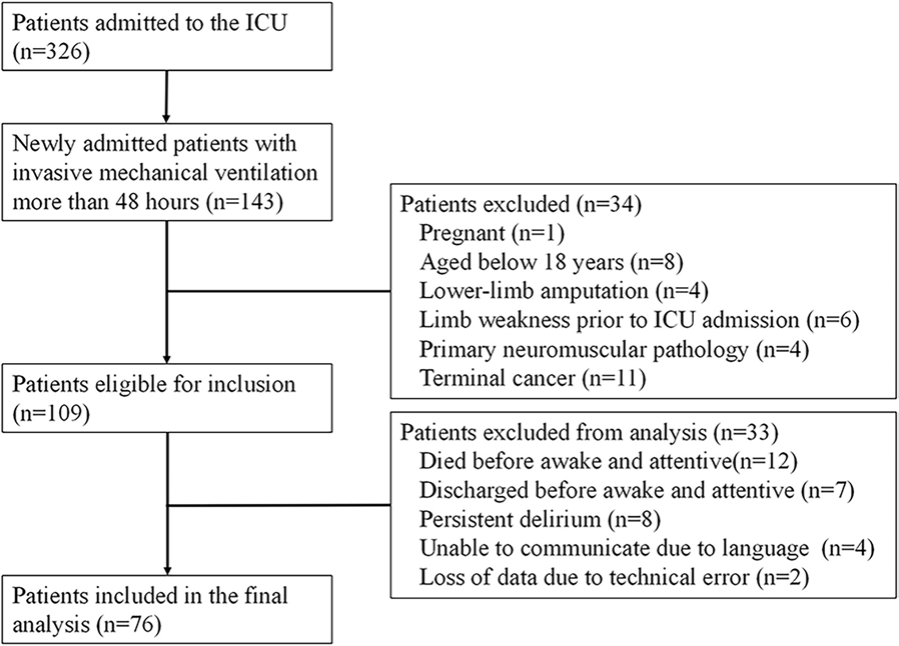
Flow diagram of patients screening and inclusion in the study

The flowchart illustrating the process of selecting the study participants is shown in Fig. 2. Between December 1, 2022, and July 1, 2023, a total of 326 critically ill patients consecutively admitted to the ICU were screened for inclusion in this study. Among them, 76 patients who met the criteria of receiving invasive MV for more than 48 h without any exclusion criteria were included in the final analysis.

### Factors associated with ICUAW

The baseline characteristics of the patients are presented in Table 1. Among the 76 patients included in the study, 34 (44.7%) were diagnosed with ICUAW based on an MRC score less than 48. Patients with ICUAW were found to have a higher proportion of sepsis (61.8% vs. 11.9%, *p* < 0.001) and lower levels of albumin at ICU admission (23.7 ± 3.2 g/L vs. 30.0 ± 4.2 g/L, *p* < 0.001) than those without ICUAW. Additionally, they had a longer median length of ICU stay (10.8 ± 7.3 days vs. 4.5 ± 1.8 days, *p* < 0.001) and longer duration of deep sedation (5.1 ± 3.0 days vs. 1.5 ± 1.3 days, *p* < 0.001) before MRC scoring. Patients with ICUAW also exhibited a lower incidence of tight glycemic control (23.5% vs. 45.2%, *p* = 0.049) and higher daily norepinephrine doses (8.1 ± 5.8 mg/day vs. 2.4 ± 2.1 mg/day, *p* < 0.001). Furthermore, patients with ICUAW needed a longer length of ICU stay (17.4 ± 16.0 days vs. 11.0 ± 9.2 days, *p* = 0.033) and a longer duration of mechanical ventilation (12.1 ± 11.3 days vs. 5.6 ± 4.5 days, *p* < 0.001) and exhibited a more pronounced rate of RFCSA atrophy between D1 and D3 (7.9 ± 2.8% vs. 4.3 ± 2.1%, p < 0.001).

**Table 1:** Comparison of patients with ICUAW and non-ICUAW determined by MRC score

Variables	ICUAW (n = 34)	No-ICUAW (n = 42)	p value
Characteristics of the patients at ICU admission			
Age, mean(SD), years	62.1(16.6)	59.4(15.6)	0.363
Male sex, No.(%)	22 (64.7)	27 (64.3)	0.970
Body mass index, mean(SD), kg/m^2^	22.5(3.8)	22.4(3.4)	0.979
Type of admission			0.737
Elective surgery, No.(%)	4(11.8)	3(7.1)	
Emergency surgery, No.(%)	3(8.8)	5(11.9)	
Nonsurgery, No.(%)	27(79.4)	34(81.0)	
APACHE II score at admission, mean(SD)	23.6(5.1)	21.0(7.0)	0.066
Sepsis, No.(%)	21(61.8)	5(11.9)	< 0.001
Albumin, mean(SD), g/L	23.7(3.2)	30.0(4.2)	< 0.001
Blood lactate, mean(SD), mmol/L	1.8(1.1)	2.7(3.7)	0.826
Comorbidities			
Hypertension, No.(%)	10(29.4)	12(28.6)	0.936
Congestive heart failure, No.(%)	11(32.4)	15(35.7)	0.759
Diabetes, No.(%)	5(14.7)	7(16.7)	0.816
Chronic obstructive pulmonary disease, No.(%)	15(44.1)	16(38.1)	0.595
Chronic kidney disease (estimated GFR < 60), No.(%)	6(17.6)	10(23.8)	0.512
Cardiac arrhythmia, No.(%)	8(23.5)	14(33.3)	0.349
Characteristics at time of MRC assessment			
MRC score, mean(SD), points	28.0(11.6)	51.6(3.5)	< 0.001
Length of ICU stay before MRC scoring, mean(SD), days	10.8(7.3)	4.5(1.8)	< 0.001
Duration of sedation before MRC scoring, mean(SD), days	6.5(3.5)	5.2(2.9)	0.081
Duration of deep sedation before MRC scoring, mean(SD), days	5.1(3.0)	1.5(1.3)	< 0.001
Average norepinephrine dosage, mean(SD), mg/day	8.1(5.8)	2.4(2.1)	< 0.001
Early parenteral nutrition during the first week of ICU, No.(%)	17(50.0)	16(38.1)	0.298
Tight glycemic control *, No.(%)	8(23.5)	19(45.2)	0.049
Outcomes			
Length of ICU stay, mean(SD), days	17.4(16.0)	11.0(9.2)	0.033
Duration of mechanical ventilation, mean(SD), days	12.1(11.3)	5.6(4.5)	< 0.001
RFCSA atrophy rate between D1 and D3, mean(SD), %	7.9(2.8)	4.3(2.1)	< 0.001

^*^tight glycemic control was aimed at blood glucose levels of between 80 and 110 mg/dl (target glucose of 80–110 mg/dl) using an insulin infusion with subcutaneous continuous glucose monitoring. ICU: Intensive care unit; ICUAW: ICU-acquired weakness; MRC: Medical Research Council; SD: standard deviation; APACHE II: Acute Physiology and Chronic Health Evaluation II; GFR: glomerular filtration rate; RFCSA: rectus femoris cross-sectional area

### Spearman correlation analysis

**Fig. 3:**
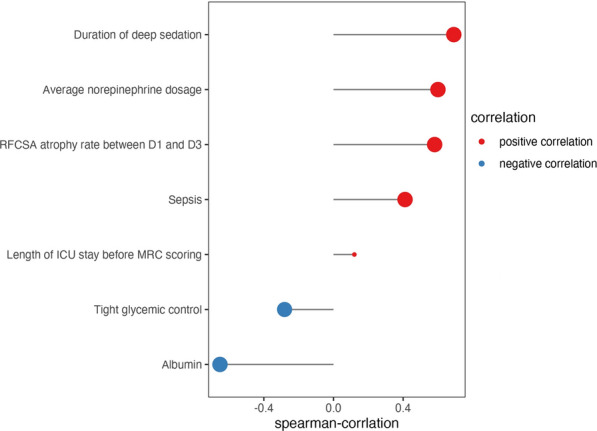
Results of Spearman correlation analysis

The results of Spearman correlation analysis indicated positive correlations between ICUAW and several factors, including the duration of deep sedation before MRC scoring, average norepinephrine dosage, RFCSA atrophy rate between D1 and D3, occurrence of sepsis, and length of ICU stay before MRC scoring. The corresponding correlation coefficients for these factors were 0.691 (*p* < 0.001), 0.600 (*p* < 0.001), 0.581 (*p* < 0.001), 0.411 (*p* < 0.001), and 0.120 (*p* = 0.303), respectively. In contrast, tight glycemic control before MRC assessment and albumin levels upon admission exhibited negative correlations with the progression of ICUAW, with correlation coefficients of − 0.281 (*p* = 0.014) and − 0.652 (*p* < 0.001), respectively, as illustrated in Fig. 3.

### Diagnostic value of ultrasound measurements of RFCSA in predicting ICUAW

**Fig. 4:**
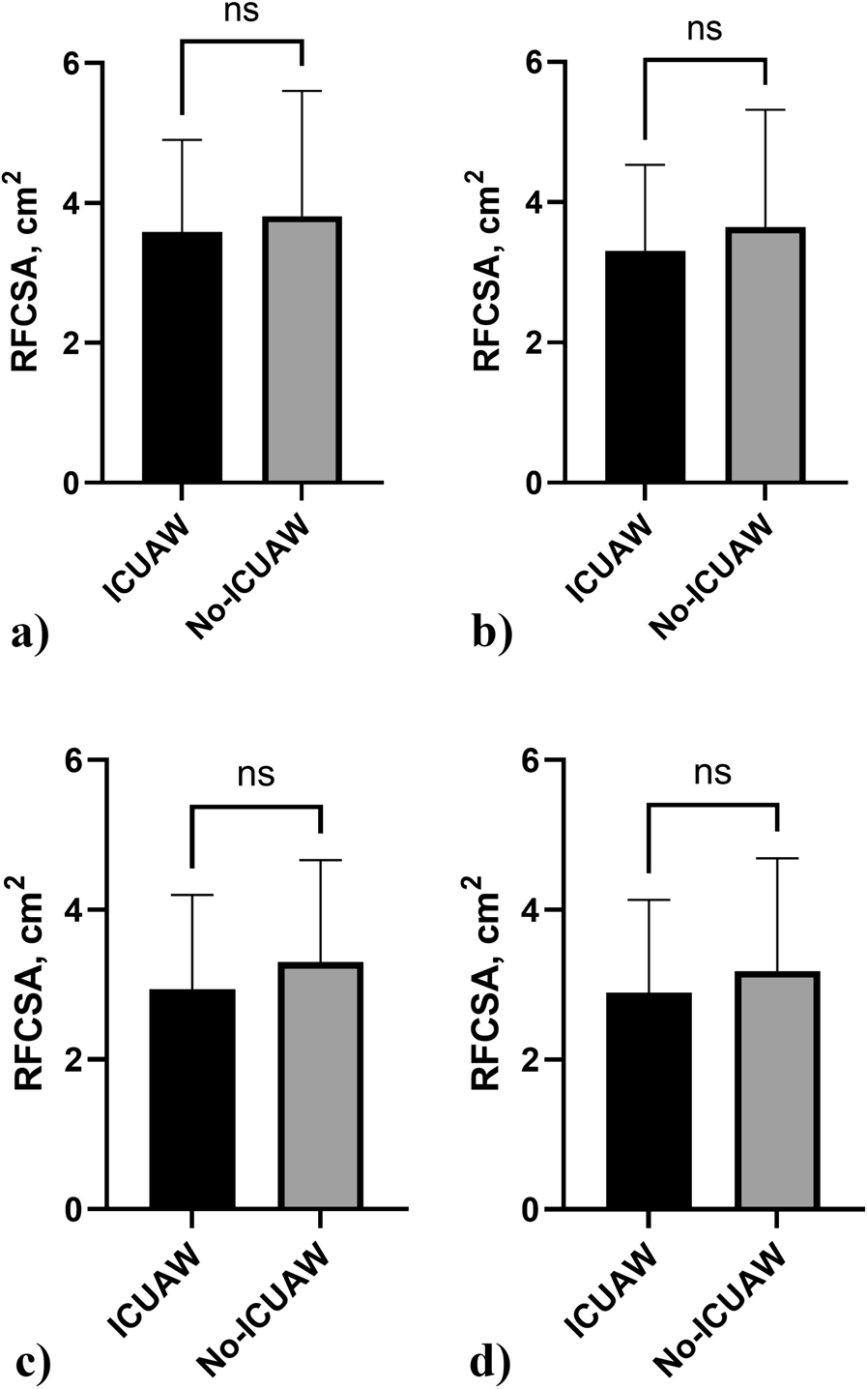
Box plots illustrating the RFCSA data comparison between ICUAW and No-ICUAW patients at four time points: ICU admission (**a**), D3 (**b**), D5 (**c**), and D7 (**d**)

**Fig. 5:**
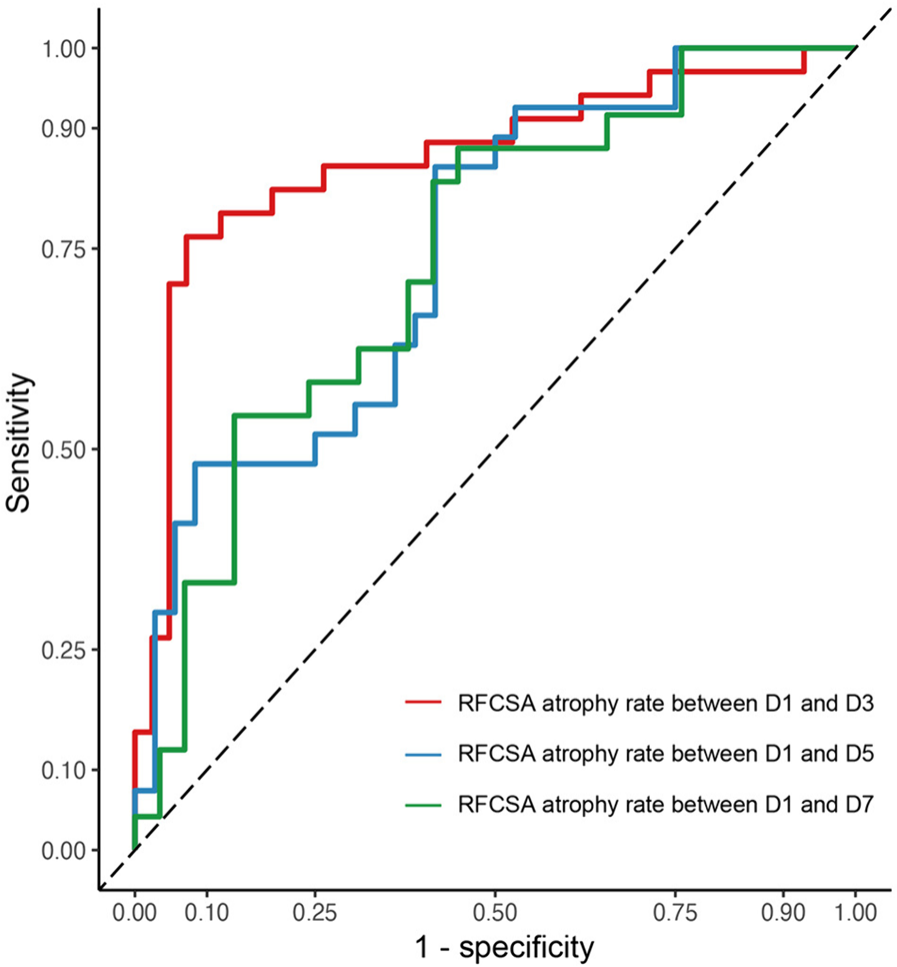
ROC curve for diagnosing ICUAW using RFCSA atrophy rate determined by ultrasound measurements. ROC, receiver operating characteristic; ICUAW, ICU-acquired weakness

The RFCSA exhibited a continuous decline subsequent to patients' admission to the ICU. Box plots were used to compare RFCSA data between ICUAW and No-ICUAW patients at four time points: ICU admission (D1), D3, D5, and D7. The findings revealed the following results: 3.59 ± 1.31 cm^2^ vs 3.81 ± 1.79, *p* = 0.541; 3.31 ± 1.22 cm^2^ vs 3.64 ± 1.67, *p* = 0.335; 2.94 ± 1.26 cm^2^ vs 3.31 ± 1.36, *p* = 0.276; 2.89 ± 1.24 cm^2^ vs 3.18 ± 1.50, *p* = 0.454 (Fig. 4). Upon assessing the absolute RFCSA values, no statistical significance was observed between the patient groups. Figure 5 displays the ROC curves for predicting the likelihood of ICUAW based on the RFCSA atrophy rate between D1 and D3, between D1 and D5, and between D1 and D7. The cutoff values for these factors were determined to be 6.9%, 8.7%, and 14.5%, respectively. Among these factors, the RFCSA atrophy rate between D1 and D3 exhibited the highest diagnostic accuracy in predicting ICUAW, with an area under the ROC curve (AUC) value of 0.861 (*p* < 0.05). It demonstrated a sensitivity of 76.5% and specificity of 92.9%. The RFCSA atrophy rate between D1 and D5 ranked second in diagnostic accuracy (AUC = 0.749, *p* < 0.05), with a sensitivity of 85.2% and specificity of 58.3%. Conversely, the RFCSA atrophy rate between D1 and D7 had the lowest diagnostic value (AUC = 0.739, *p* < 0.05), with a sensitivity of 87.5% and specificity of 55.2%.

### Exploratory analysis of outcomes in mechanically ventilated patients

**Fig. 6:**
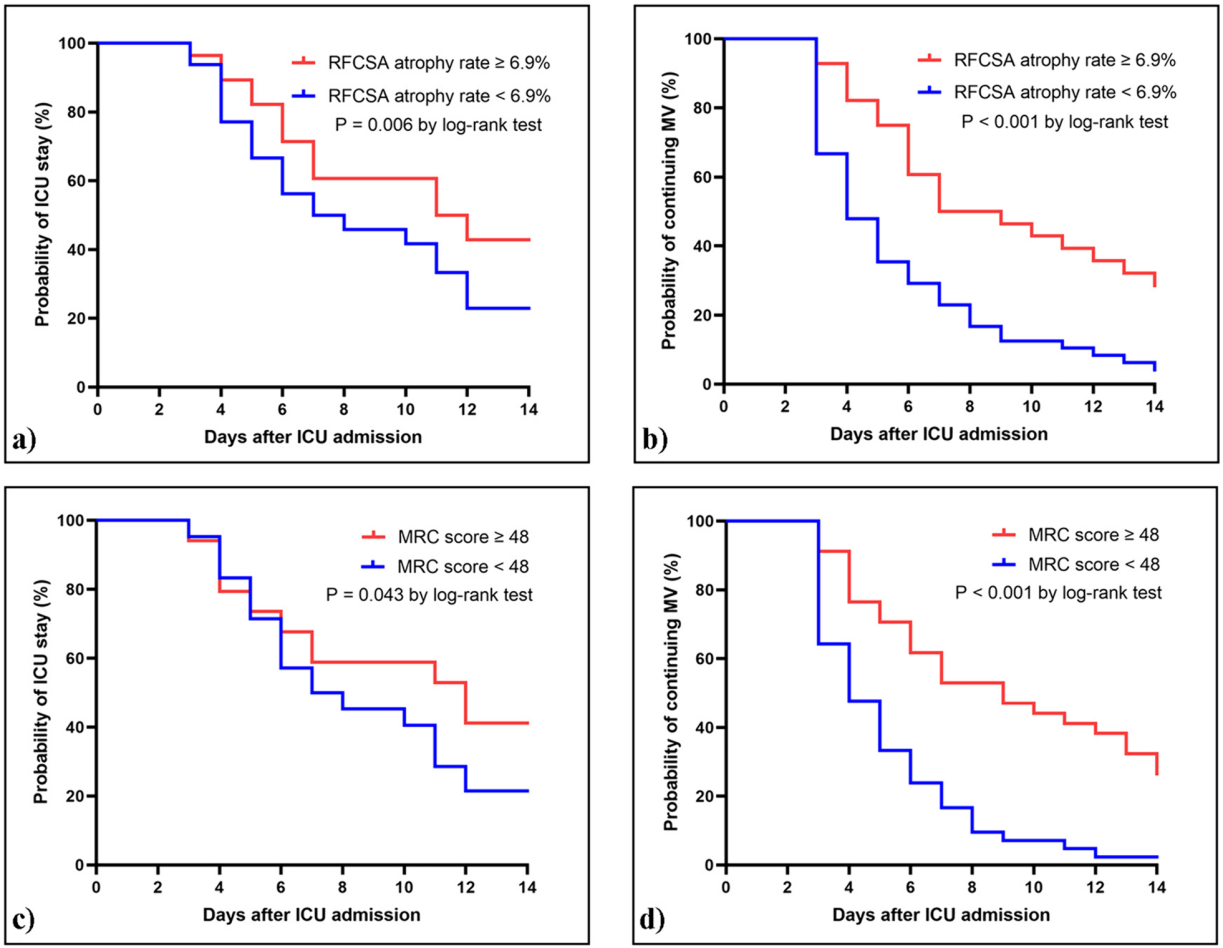
Clinical outcomes of patients diagnosed with ICUAW by ultrasound measurements and MRC score. Kaplan‒Meier analysis conducted to assess ICU stay (**a**) and continuing MV (**b**) probability in patients with and without ICUAW determined by RFCSA atrophy rate between D1 and D3. The red line represents patients with RFCSA atrophy rate between D1 and D3 ≥ 6.9% (n = 28), while the blue line represents patients with RFCSA atrophy rate between D1 and D3 < 6.9% (n = 48). Kaplan‒Meier analysis conducted to assess ICU stay (**c**) and continuing MV (**d**) probability in patients with and without ICUAW determined by MRC score. The red line represents patients with MRC score ≥ 48 points (n = 34), while the blue line represents patients with MRC score < 48 points (n = 42). MV, mechanical ventilation; MRC, Medical Research Council

Furthermore, a significant difference was observed between the groups in terms of the length of ICU stay and duration of invasive MV. In patients with ICUAW diagnosed based on an RFCSA atrophy rate between D1 and D3 ≥ 6.9%, 42.9% (12 out of 28 patients) had an ICU stay longer than 14 days compared to 22.9% (11 out of 48 patients) in those without ICUAW. Kaplan–Meier analysis indicated that patients in the ICUAW group experienced a delayed ICU stay during hospitalization compared to patients without ICUAW (HR: 1.768; 95% CI 1.128–2.772; *p* = 0.006; Fig. 6a). Moreover, ICUAW was significantly associated with a delayed time to discontinuation MV. The proportion of patients continuing MV at 14 days was 28.6% versus 4.2% between the two groups (HR: 1.988; 95% CI 1.266–3.120; *p* < 0.001; Fig. 6b). For ICUAW patients diagnosed by MRC score, the proportion of patients with an ICU stay longer than 14 days was 41.2% (14 out of 34 patients) compared to 21.4% (9 out of 42 patients) in the non-ICUAW group (HR: 1.525; 95% CI 0.970–2.397; *p* = 0.043; Fig. 6c). Similarly, the proportion of patients continuing MV at 14 days was 23.5% versus 2.4% between the two groups (HR: 2.160; 95% CI 1.354–3.447; *p* < 0.001; Fig. 6d).

### Multivariate analysis

According to the multivariate logistic regression model, albumin at ICU admission, duration of deep sedation, and average norepinephrine dosage before MRC scoring were found to be the three variables independently associated with ICUAW. The adjusted odds ratios were − 0.416 (95% CI 0.448–0.971, *p* = 0.035), 1.366 (95% CI 1.026–14.991, *p* = 0.046), and 0.574 (95% CI 1.053–2.993, *p* = 0.032), respectively. It was observed that lower albumin levels at ICU admission were associated with a greater risk of developing ICUAW. Other factors, including length of ICU stay or duration of MV before MRC scoring, were included in the model; however, they did not show statistical significance.

## Discussion

In this prospective cohort study, which included data from 76 participants, the ultrasound measurements revealed a decrease in the percentage of RFCSA by 7.9 ± 2.8% and 4.3 ± 2.1% from the first to the third day among patients with and without ICUAW, respectively. We observed that patients with greater muscle loss experienced longer durations of ICU stay and invasive MV. Additionally, this study demonstrated that ultrasound measurements of the rectus femoris muscle had high diagnostic accuracy in identifying ICUAW in patients who required invasive MV. We also observed a positive association between ICUAW and various factors. These factors included albumin at ICU admission, the duration of deep sedation before MRC scoring, RFCSA atrophy rate between D1 and D3, average norepinephrine dosage, length of ICU stay before MRC scoring, and the occurrence of sepsis. After adjusting for several confounding factors, albumin at ICU admission, the duration of deep sedation, and average norepinephrine dosage before MRC scoring remained significant.

There are currently some different theories on the pathophysiological mechanisms of skeletal muscle atrophy in critically ill patients. The balance between muscle protein synthesis and proteolysis in critically ill patients is disrupted, favoring catabolism. This shift toward catabolism occurs due to various stressors, such as sepsis, acute illness, trauma, and surgery. The ubiquitin proteasome system serves as a key regulator of muscle proteolysis, and emerging evidence suggests that its activity is heightened during the acute phase of muscle loss in critically ill patients [32]. Activation of the ubiquitin proteasome system is triggered by various upstream stimuli, oxidative and energetic stress, and mechanical silencing. These stimuli attract downstream signaling partners that specifically focus on activating the ubiquitin proteasome system [33]. Inhibition of protein synthesis is another important cause of muscle loss in critically ill patients. Protein synthesis decreases when muscles are inactive. As a critical and central promoter of protein synthesis, mammalian target of rapamycin-1 acts by regulating mRNA translation and is partially upregulated upstream of the protein kinase B signaling network, which in turn is regulated by nutrition, positive energy balance and growth factors [32]. Additionally, passive muscle stretching and other stimuli can activate mammalian target of rapamycin-1 independently of protein kinase B. Previous studies have demonstrated that glucocorticoids can exacerbate muscle wasting in septic patients. These actions contribute to the degradation of muscle proteins and aggravate muscle wasting.

Over the past decade, ultrasound has increasingly gained prominence in ICU patient management and has recently been proposed as a tool for measuring muscle characteristics such as cross-sectional area [28], thickness, and echo intensity [22, 34]. There is still uncertainty regarding the effectiveness of ultrasound measurements in assessing RFCSA. A recent study conducted on long-term critically ill patients, with an expectation ICU stay of at least 72 h, reported no significant differences in the percent change of RFCSA over time [28]. However, the study is limited by inclusion of only a small sample size, single-center study design, and focus on a single type of disease. This study primarily enrolled critically ill medical patients who typically present with chronic underlying diseases and suboptimal nutritional status. These patients often experience a persistent negative protein balance, leading to muscle breakdown and consequently exhibit relatively low baseline RFCSA levels. Consequently, the visible manifestation of muscle atrophy may not be immediately apparent within the initial days following admission to the ICU. However, it should be noted that thickness measurements tend to underestimate muscle wasting in the ICU when compared to RFCSA measurements. Previous studies have emphasized the significance of RFCSA measurement as a biomarker for assessing muscle loss in proximal lower-limb muscles and knee extensor weakness during the early stages of critical illness [20, 35]. In terms of diagnosing ICUAW, ultrasound measurements of muscle atrophy rates have demonstrated good accuracy when compared to reference methods [35]. Hence, ultrasound assessment holds promise as a valuable bedside tool for immediate and reproducible muscle measurements. Various studies have indicated that muscle mass declines rapidly in the ICU setting, and a 10% decline in RFCSA during the first week of ICU stay is associated with an increased number of organ failures, prolonged mechanical ventilation, and a higher incidence of post-critical care weakness and restriction [16]. The likely explanation lies in the acute phase of critical illness, which comprises two distinct periods: an early phase characterized by metabolic instability and a significant increase in catabolism, leading to substantial muscle loss predominantly; and a later phase marked by pronounced muscle wasting and the stabilization of metabolic disruptions. Malnutrition and muscle wasting often become evident during ICU stays due to the influence of catabolic hormones, dietary imbalances, and prolonged physical immobility. Considerable reductions in muscle mass and fat mass can occur within a relatively short timeframe in the ICU environment. Critically ill patients undergo declines in muscle mass, strength, endurance, and mobility, making them remarkably akin to typically frail, geriatric individuals [36]. These findings are partially in line with our own research results.

Previous studies have indicated that assessing muscle parameters at a single time point holds little significance in identifying ICUAW. However, it is worth considering that the magnitude of changes in these parameters may have diagnostic value [22]. In our study, we observed a significant reduction in the cross-sectional area of the rectus femoris muscle on D3 after admission to the ICU for patients with ICUAW compared to those without ICUAW. Building upon this observation, we further explored the diagnostic potential of muscle atrophy rate for ICUAW. Our results demonstrate that the atrophy rate of RFCSA has high diagnostic value. Specifically, on D3 of ICU admission, a decrease of 6.9% in the cross-sectional area of the rectus femoris can serve as an early identifier of ICUAW patients, with an AUC of 0.861, indicating strong diagnostic value. Muscle ultrasound shows great potential in detecting ICUAW patients and offers a novel method for early clinical diagnosis of ICUAW. This is particularly valuable when patients cannot be evaluated using MRC scoring, as assessing changes in muscle cross-sectional area through ultrasound can enable the identification of ICUAW patients and facilitate early intervention.

### Limitations

In our study, we aimed to assess the diagnostic accuracy of muscular ultrasonographic studies at various time points in patients using the MRC score as a reference standard. Since muscle biopsy or electrophysiological recordings were not routinely conducted in our ICU clinical practice, we were unable to differentiate between critical illness myopathy, critical illness polyneuropathy, and critical illness neuromyopathy. Furthermore, in this study, there was a lack of synchronization between muscular ultrasonographic assessments and strength evaluations. The assessments of strength were generally delayed in patients with ICUAW compared to those without ICUAW. This discrepancy in timing may have potentially exaggerated the differences observed between the two groups, as patients with ICUAW had more time for muscle cross-sectional area and strength to decrease. Additionally, the limited number of blind dual-operator assessments represents another limitation of this study. Although the operators performing ultrasound measurements were blinded to the MRC scores, complete blinding to muscle strength is not feasible since the presence or absence of spontaneous movements already provides an impression of muscle strength. Besides, to ensure that the muscle was fully represented in the ultrasound image, the operator used minimal force on the leg, although this could have caused distortion of the muscle and affected the cross-sectional area. However, if the muscle was not fully visible in the ultrasound image, it may have been necessary to estimate the boundaries of the muscle, resulting in a potentially greater error in measuring the muscle's cross-sectional area. The potential bias in the study results is also linked to the utilization of sedative medications. Drawing a clear distinction between the impacts of sedatives and those resulting from immobility induced by sedation and prolonged bed rest poses considerable challenges. It is believed that maintaining sedation exerts a more significant influence on both muscle atrophy and weakness compared to scenarios where a patient remains awake but immobilized without sedation [2]. Therefore, it is crucial to validate the results presented in our study through larger-scale investigations. Future studies should be designed to address the limitations mentioned above and provide a more comprehensive understanding of the diagnostic accuracy of ultrasonographic assessments in ICUAW patients.

## Conclusions

Our study shows that a decrease of more than 6.9% in RFCSA within the first three days of ICU admission effectively identifies patients with ICUAW and indicates a longer ICU stay and an extended duration of invasive MV. The ultrasound measurements of RFCSA proves to be a precise and practical bedside method for diagnosing ICUAW and indicating prognosis in critically ill patients receiving invasive MV.

## Data Availability

All relevant data and materials have been involved in the article. Further inquiries can be directed to the corresponding authors.

## Abbreviations

ICU: Intensive care unit
ICUAW: ICU-acquired weakness
RFCSA: Rectus femoris cross-sectional area
MV: Mechanical ventilation
MRC: Medical Research Council
APACHE II: Acute Physiology and Chronic Health Evaluation II
D: Day
SD: Standard deviation
HR: Hazard ratios
CI: Confidence intervals
ROC: Receiver operating characteristic
AUC: Area under the ROC curve
